# Towards a Methodology for Validation of Centrality Measures in Complex Networks

**DOI:** 10.1371/journal.pone.0090283

**Published:** 2014-04-07

**Authors:** Komal Batool, Muaz A. Niazi

**Affiliations:** 1 National University of Science & Technology, Islamabad, Pakistan; 2 Bahria University, Islamabad, Pakistan; 3 COSIPRA Lab, University of Stirling, Stirling, Scotland, United Kingdom; University of Maribor, Slovenia

## Abstract

**Background:**

Living systems are associated with Social networks — networks made up of nodes, some of which may be more important in various aspects as compared to others. While different quantitative measures labeled as “centralities” have previously been used in the network analysis community to find out influential nodes in a network, it is debatable how valid the centrality measures actually are. In other words, the research question that remains unanswered is: how exactly do these measures perform in the real world? So, as an example, if a centrality of a particular node identifies it to be important, is the node actually important?

**Purpose:**

The goal of this paper is not just to perform a traditional social network analysis but rather to evaluate different centrality measures by conducting an empirical study analyzing exactly how do network centralities correlate with data from published multidisciplinary network data sets.

**Method:**

We take standard published network data sets while using a random network to establish a baseline. These data sets included the Zachary's Karate Club network, dolphin social network and a neural network of nematode Caenorhabditis elegans. Each of the data sets was analyzed in terms of different centrality measures and compared with existing knowledge from associated published articles to review the role of each centrality measure in the determination of influential nodes.

**Results:**

Our empirical analysis demonstrates that in the chosen network data sets, nodes which had a high Closeness Centrality also had a high Eccentricity Centrality. Likewise high Degree Centrality also correlated closely with a high Eigenvector Centrality. Whereas Betweenness Centrality varied according to network topology and did not demonstrate any noticeable pattern. In terms of identification of key nodes, we discovered that as compared with other centrality measures, Eigenvector and Eccentricity Centralities were better able to identify important nodes.

## Introduction

Living systems are associated with Social networks — networks involve diffusion of information from one node to the other, some of which may be more important than others. While different quantitative measures labeled as “centrality” measures have previously been used in the network analysis community to find out influential nodes in a network, it is debatable how valid the centrality measures actually are. In other words, the research question that remains unanswered is: how exactly do these measures correlate with the real world? After all, the real world is not based on just the network ties. Besides circumstances can change the importance of any given node. So, as an example, will a node always remain influential and important in the real world just because it occupies a more central location in a given network?

On its face, the problem may not appear to be grave. However, in practice this can be a very serious problem especially when social network methods are used on actual human beings — such as for the detection of malicious individuals in air travel. Obviously, in such situations, false negatives can be extremely detrimental because they would imply that a malicious individual was able to board an aircraft without being detected by the system. Whereas, false positives can result in serious economic problems in air travel thereby not only wasting valuable time as well as resources while falsely characterizing people and having them extensively searched due to results from black-box algorithms and probabilistic models — essentially based on concepts from mathematical models such as centralities. Thus, there certainly exists a need to test the efficacy and validity of individual centrality measures to correctly identify influential nodes in networks.

The goal of this paper is not just to perform a traditional social network analysis but rather to evaluate the validity of different centrality measures by conducting an empirical study analyzing the correlation of various network centralities with real-world data from published multidisciplinary network data sets. Additionally, we present first steps towards developing a formal methodology for the validation of centrality measures by demonstrating how to perform validation of centrality measures in a given network. By examination and correlation of several different commonly used centrality measures, we believe this study serves as an example lays out first steps for conducting similar studies for the identification of relatively stronger candidates among the centrality measures for a given data set — centralities which are more capable of predicting real-world important and more central nodes. While we realize that the study itself may not decisively prove that the same measures may always be important likewise in any given empirical network, it does however lay grounds for further studies in the same context.

We take standard published network data sets in addition to a random network as a baseline. These data sets included the Zachary's Karate Club network, dolphin social network and a neural network of nematode Caenorhabditis elegans. Each of the data sets was analyzed in terms of different centrality measures and compared with existing knowledge about important nodes from associated published literature to review the role of each centrality measure in the determination of influential nodes. The peculiar goal of this paper required the use of standard and relatively smaller published data sets in contrast to larger, unpublished data sets because the goal of this study is not just to perform a social network analysis or present a particular network data set — which would also not have been exciting. The reason for choosing these particular data sets was that these have already been examined by the community of network researchers and thus there is existing published information available about them. Additionally, for a more general applicability, we also ensured the use of different types of data sets rather than only considering human social networks.

Our empirical analysis demonstrates that, in our chosen data sets, nodes which have a high Closeness Centrality also had a high Eccentricity Centrality. Likewise high Degree Centrality also correlated closely with a high Eigenvector Centrality. Whereas Betweenness Centrality varied according to network topology and did not demonstrate any similar noticeable pattern. In terms of identification of key nodes, we have discovered that as compared with other centrality measures, Eigenvector as well as Eccentricity Centralities were better able to identify important nodes.

The outline of the rest of the paper is as follows:

We first present background about networks and centralities. Next, in the methodology section, we discuss the data sets and the centralities analyzed in the networks. Then in the results section, we discuss the implications of analyzing the network using centralities in the networks. This is followed by conclusions and future work section.

## Background

Networks allow for modeling complex interactions of components in the form of a standard set of representations [25]. These representations can be used to model a wide range of complex systems — systems as diverse and ranging from those involving the co-expression of genes to interaction of online peers in a peer-to-peer file sharing network or humans connecting together in a social community to animals communicating and interacting with each other [9]. In all such networks, a key dynamical process is the fact that each network spreads some quantity of information from one node to the other. This information can again be quite diverse ranging from the amount of disease spread between connected cities to loss of personal information and privacy in online social networks such as Twitter, Facebook, LinkedIn or Google+. For the purpose of analysis of nodes which may be influential in these networks, various quantitative measures (or centralities) have previously been devised to identify the key nodes in the network. Generally a social network is a group of interconnected social entities such as individuals or organizations. The growth of Internet and World Wide Web has enabled us to study large-scale social networks due to an exponentially growing interest in social network analysis [23], [21], [18]. It is pertinent to note here that networks have previously been described as an alternative approach to modeling these Complex Adaptive Systems (CAS) [26], in addition to agent-based [24].

The critical position of a node in a network is considered by many as a function of its centrality. However, Bampo et al. [2] notes in contrast to this opinion that that the flow of information in networks is affected not just by the network structure (marked by centrality) but actually by three major factors:

1. Network structure as marked by its centrality [13]
2. Behavioral characteristics of these members
3. Information attributes

Studies such as by Newman [22] have used simulations on different random and real networks to study the influence of social power by considering the degrees of the nodes on the development of continuous opinions in complex networks by employing numerical simulations.

However, researchers such as Barabasi have identified the importance of studying the temporal nature of network dynamics such as in the form of “hot spots” [3]. Likewise, we believe that typical networks can be considered as a snapshot of real-world networks and the typical centrality measures alone do not suffice to capture these more complex dynamics which are hidden inside or from the real-world network. It is these differences which need to be examined in more detail with the current paper serving as a first step in this direction.

Studies such as by Newman [22] have employed simulations on different random and real networks to study the influence of social power by considering the degrees of the nodes on the development of continuous opinions in complex networks by employing numerical simulations.

However, researchers such as Barabasi have identified the importance of studying the temporal nature of network dynamics such as in the form of “hot spots” [3]. Likewise, we believe that typical networks can be considered as a snapshot of real-world networks and the typical centrality measures alone do not suffice to capture these more complex dynamics which are hidden inside or from the real-world network. It is these differences which need to be examined in more detail with the current paper serving as a first step in this direction.

## Methodology

**Figure 1:**
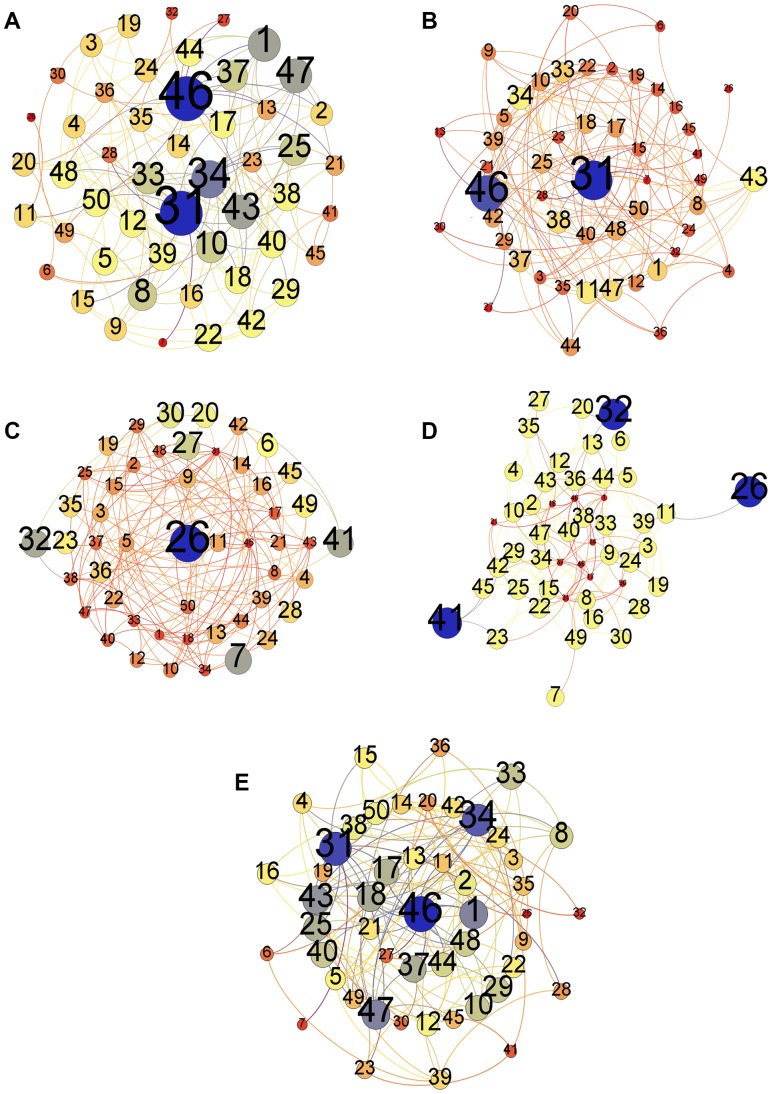
Methodology Pipeline. (a) Degree Centrality. (b) Betweenness Centrality. (c) Closeness Centrality. (d) Eccentricity Centrality. (e) Eigenvector Centrality.

**Figure 2:**
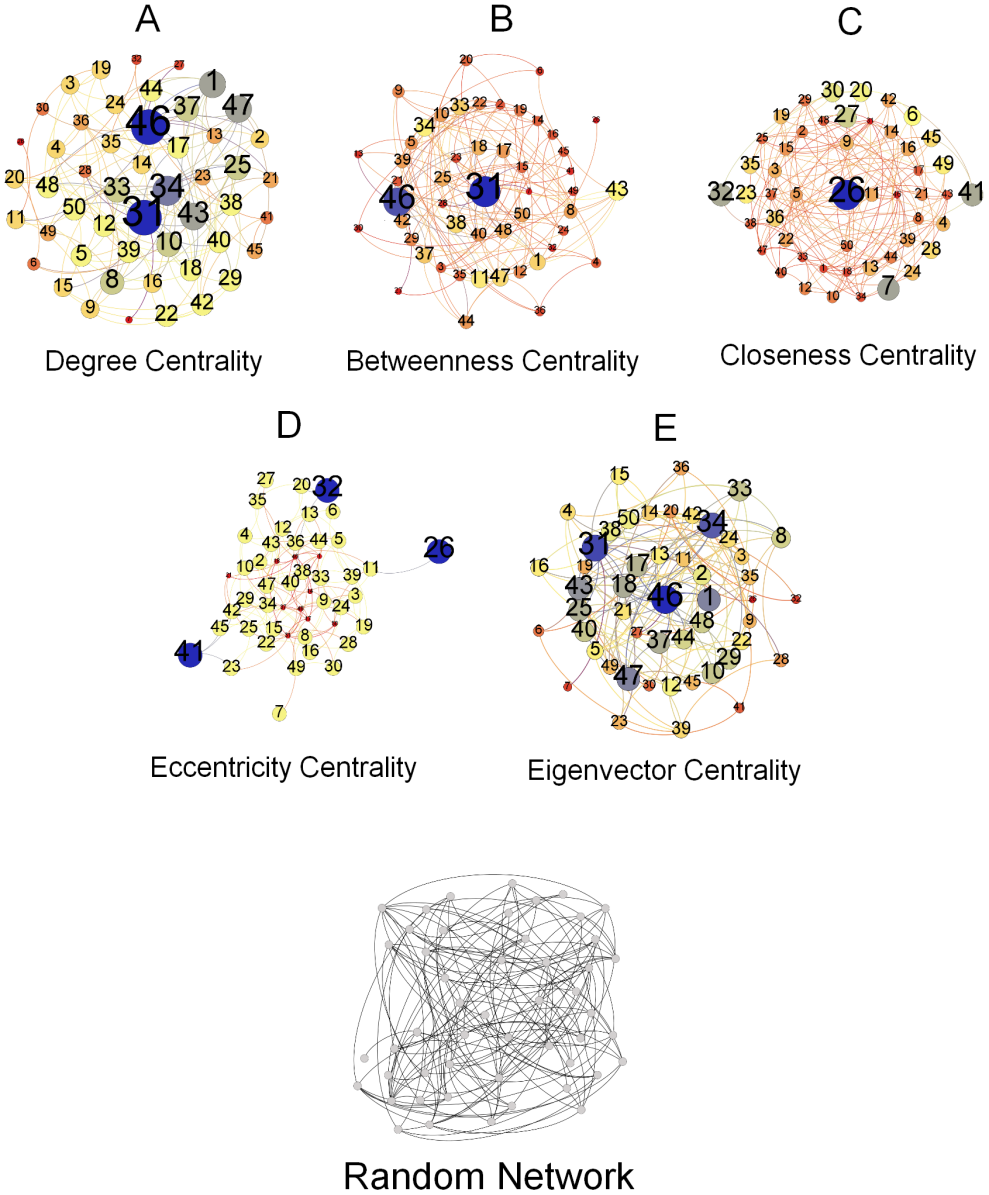
Erdős–Rényi (Random) Network. Figure 2 represents the Erdös–Rnyi network formed with the *p* = 0.1. The network consists of a source, target and intermediate laid randomly in the network. Figure 2a represents the degree centrality of the individual nodes according to the size and color variation. Nodes (blue) have the highest degree centrality and thus have the largest size in the network where as the nodes (red) have the smallest value of degree centrality in the network. Figure 2b represents betweenness centrality of the nodes in the network. Nodes (blue) have the highest betweenness centrality and have the largest size in the network as the betweenness value decreases so the size and also the color changes ultimately to red. Figure 2c and figure 2d represents closeness centrality and eccentricity centrality of nodes of this network. Both of the centralities are analyzed on this network, the highest value nodes are represented as the largest nodes in blue color. To see the central node in the network or to observe which node is most eccentric in the network, reciprocal of these values is taken. Here, smaller the size of a node is more central and eccentric in the network. Figure 2e represents the eigenvector centrality of the nodes in the network. The highest value nodes are represented in blue color where as nodes with lowest values are represented in red color. (a) Degree Centrality. (b) Betweenness Centrality. (c) Closeness Centrality. (d) Eccentricity Centrality. (e) Eigenvector Centrality.

**Figure 3:**
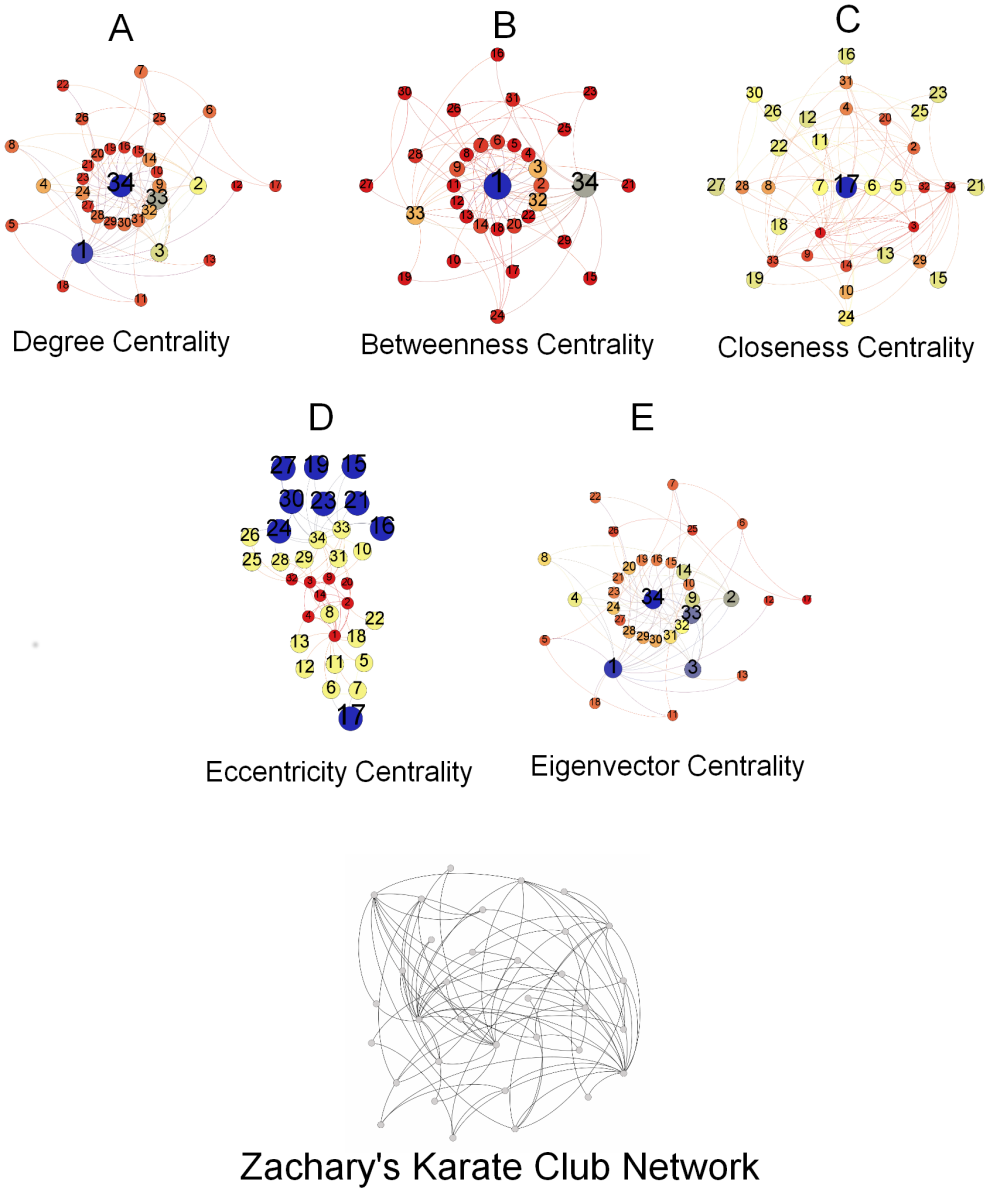
Zachary's Karate Club Network. Figure 3 represents Zachary's Karate Club network. The network is laid out randomly representing source and intermediate nodes as club instructor, club president and officers in the network. Club instructor and club president either of them is considered to be a source node of information flow in the network. Figure 3a represents the degree centrality of the individual nodes according to the size and color variations. Nodes in blue color have the highest degree centrality and thus have the largest size in the network where as nodes in red color have the least value of degree centrality. Figure 3b represents betweenness centrality of the nodes in the network. Nodes (blue) have the highest betweenness centrality and have the largest size in the network as the betweenness value decreases so the size and also the color changes ultimately to red. Figure 3c and figure 3d represents closeness centrality and eccentricity centrality respectively. Both of the centralities analyzed on the network show that the highest value nodes are represented as the largest nodes in blue color. To see the central node in the network or to observe which node is most eccentric in the network, reciprocal of these values is taken. Here, smaller the size of a node is more central and eccentric in the network figure 3e represents the eigenvector centrality of the nodes in the network. Nodes represented in blue color have the highest value where as nodes with lowest values are represented in red color. (a) Degree Centrality. (b) Betweenness Centrality. (c) Closeness Centrality. (d) Eccentricity Centrality. (e) Eigenvector Centrality.

**Figure 4:**
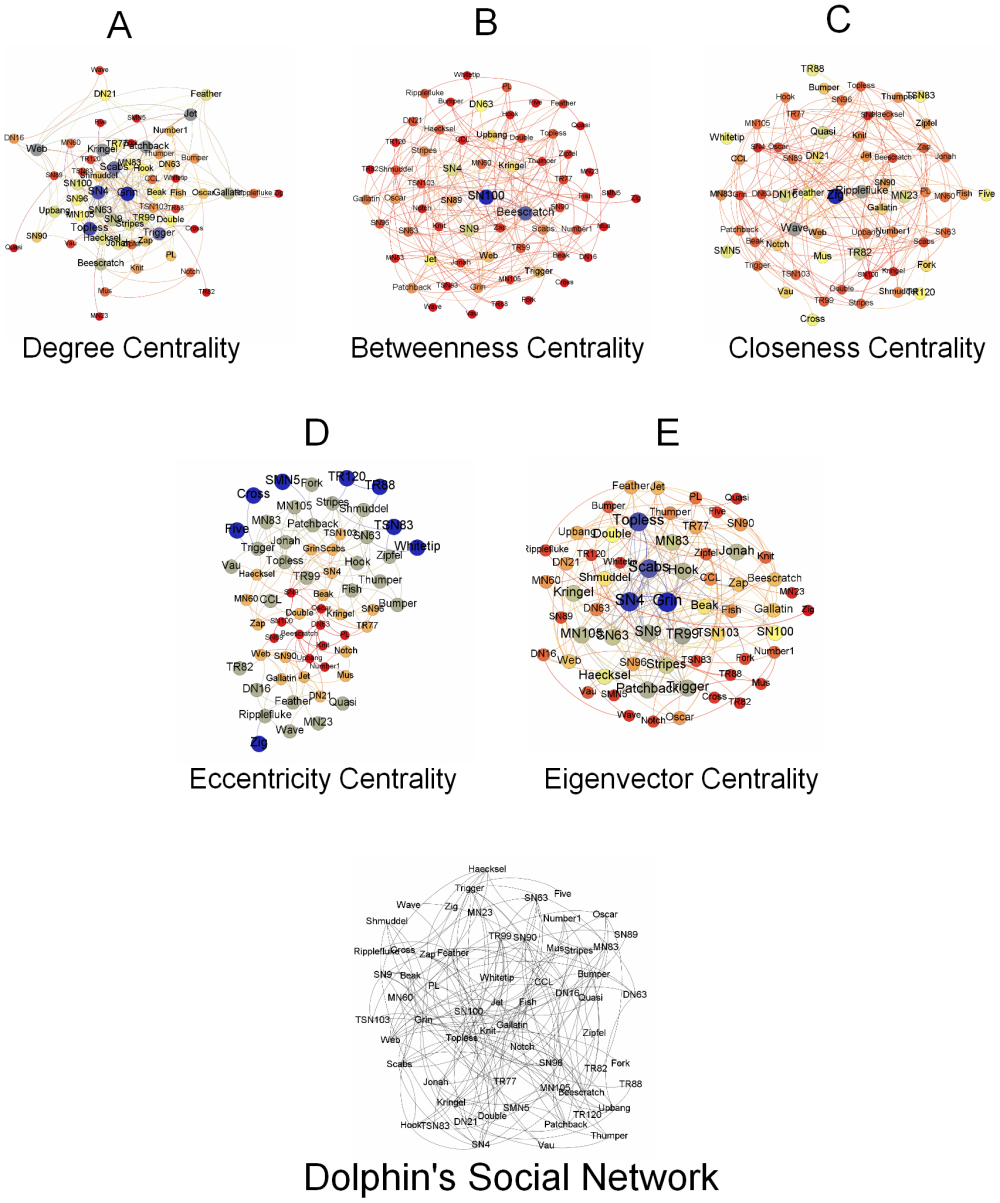
Dolphins Social Network. Figure 4 represents Dolphins social network. The details of the nodes identity are not given in the originally compiled data, therefore we assume the network laid out randomly consists of source, target and intermediate nodes. Figure 4a represents the degree centrality of the individual nodes according to the size and color variation. Nodes (blue) have the highest degree centrality and thus have the largest size in the network where as nodes (red) have the least value of degree centrality in the network. Figure 4b represents betweenness centrality of the nodes in the network. Nodes (blue) have the highest betweenness centrality and have the largest size in the network as the betweenness value decreases so the size and also the color changes ultimately to red. Figure 4c and figure 4d represents closeness centrality and eccentricity centrality of this social network. Both of the centralities analyzed for the network have the highest value nodes represented as the largest nodes in blue color. To see the central node in the network or to observe which node is most eccentric in the network, reciprocal of these values is taken. Here, smaller the size of a node is more central and eccentric in the network. Figure 4e represents the eigenvector centrality of the nodes in this social network. Nodes in blue color have the highest value of centrality where as nodes with lowest value are represented in red color. (a) Degree Centrality. (b) Betweenness Centrality. (c) Closeness Centrality. (d) Eccentricity Centrality. (e) Eigenvector Centrality.

**Figure 5:**
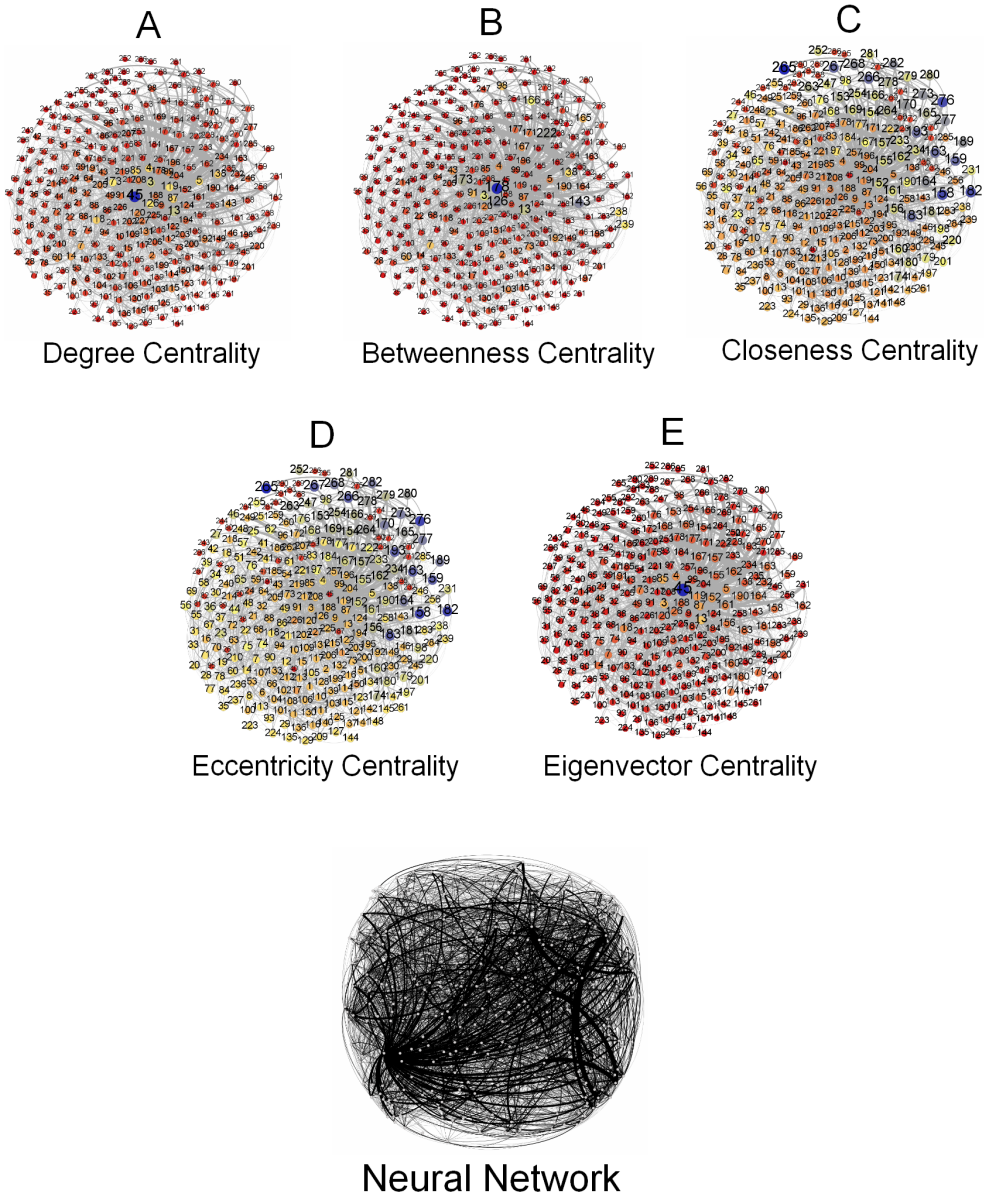
Neural Network. Figure 5 represents a neural network of nematode Caenorhabditis elegans. The details of the nodes identity are not given by the source from which the data has been collected therefore we assume the network laid out randomly consists of source, target and intermediate nodes. Figure 5a represents the degree centrality of the individual nodes according to the size and color variation. Nodes (blue) have the highest degree centrality and thus have the largest size in the network where as nodes (red) have the least value of degree centrality in the network. Figure 5b represents betweenness centrality of the nodes in the network. Nodes (blue) have the highest betweenness centrality and have the largest size in the network as the betweenness value decreases so the size and also the color changes ultimately to red. Figure 5c and figure 5d represents closeness centrality and eccentricity centrality of this neural network. Both of the centralities analyzed for the network have the highest value nodes represented as the largest nodes in blue color. To see the central node in the network or to observe which node is most eccentric in the network, reciprocal of these values is taken. Here, smaller the size of a node is more closer and eccentric in the network. Figure 5e represents the eigenvector centrality of the nodes in the network. The highest value nodes are represented in blue color where as nodes with lowest values are represented in red color. (a) Degree Centrality. (b) Betweenness Centrality. (c) Closeness Centrality. (d) Eccentricity Centrality. (e) Eigenvector Centrality.

Here we present the breakdown of our methodology in figure 1. This figure highlights the key steps undertaken in the study. We take three different data sets besides an Erdős–Rényi random network as a baseline. These networks are presented in figures 2, 3, 4, and 5. And for analyzing the key nodes in these networks, we use five centralities as shall be discussed later. As mentioned earlier, the centralities are used to highlight the importance of nodes in the networks. The breakdown of individual steps is given as follows:

1. First we generate networks from the collected data sets for random network and empirical networks.
2. Next, we apply visualization and analysis via centralities on these networks.
3. We measure each of the centralities on these simulated networks.
4. We scale the networks using these centralities and also plot centralities.
5. We then evaluate and compare each of the centralities to interpret the best centralities for measuring influential nodes on the networks.

### Data Sets

Numerous published data sets are available online as a rich source of evidence for examining the underlying formation of various networks [17] including the dynamics of individual [31] and group behavior [11], efficacy of viral product recommendation [16], global properties of email messages [34], [19], blog posts [18] as well as the identification of influential blogs [10], [18]. Many of these studies did not clearly mention the basic structure of their networks but rather had to be understood from the flow of information from one node to another. As such, for our analysis, we chose four different data sets including an Erdös–Rnyi random network [7], and 3 empirical data sets — Zachary's Karate Club Network [35], dolphins social network [20] and neural network of nematode Caenorhabditis elegans [33]. Next, we discuss the particular data sets used in the study.

### Random Network

Random network is a 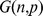 model where nodes forming a graph are connected randomly. All the edges in a graph are connected with a probability *p* where every edge is independent of other edge. Similarly, the probability of graphs having n nodes and M edges can be represented as
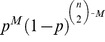
(1)Here the parameter *p*, can be considered as a weighting function; when *p* increases from 0 to 1, a graph includes more and more edges and when *p* decreases from 1 to 0, a graph becomes more and more disconnected. In particular, when 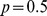, this corresponds to a case where all the n vertices of a graph are chosen with an equal probability. To serve as a baseline/comparison, we generated a 50 nodes Erdős–Rényi 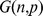 network [7] depicted in figure 2. The link probability was 0.1 with a total of 135 edges.

### Zachary's Karate Club Network

This contains a network of friendships between 34 members of Zachary's Karate Club shown in figure 3 forms 78 edges. This network is based on a study conducted at a US university described by Wayne Zachary in 1977 in [35].

### Dolphins Social Network

This social network contains the associations between 62 dolphins forming 159 edges in a community living off Doubtful Sound, New Zealand compiled by [20]. The figure 4 shows the random layout of this social network of dolphins.

### Neural Network

Figure 5 represents the random layout of the network of the nematode Caenorhabditis elegans compiled by Duncan Watts and Steven Strogatz from original experimental data done by White et al. [33]. This network contains 297 nodes and 2359 edges.

### Centralities

Freeman notes that the calculation of centrality is a key area of research focus in the domain of social network analysis research for an extended period of time [8], [15], [12]. Most commonly used centrality measures include degree centrality, closeness centrality, betweenness centrality, eccentricity centrality [6] and eigenvector centrality—with degree, closeness and betweenness measures being proposed by Freeman [8] and eigenvector centrality proposed by Bonacich [5]. Centrality is considered important by researchers because centralities formally indicate the value of nodes in the network topology. Central positions have, however, often been equated with opinion leadership or popularity [4], [27], [29], [30], [1]. Often, researchers primarily use the degree measure of centrality, perhaps because it is the easiest in terms of explanation to non-technical audiences — besides its association with behavior is intuitive. In the current paper, we are looking to evaluate and validate the role of commonly-used centralities in the identification of nodes which are actually influential in the network.

We focus on the following centralities for the analysis:

1. Degree Centrality: It is defined formally as “The number of links incident upon a node”. Degree is often considered as a means of analyzing how nodes can be affected by flow inside a given network. Directed networks can be evaluated using an in-degree and an out-degree with in-degree counting the number of links towards the node and out-degree the arcs away from it. Often links are associated with friendships — in-degree as a measure of being popular and out-degree as a metric for being gregarious. In the diffusion of information or infection, degree may translate to probabilities of receiving information or being infected” [8]. Degree centrality of a node 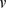 is calculated as:
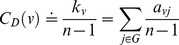
(2)where 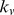 is the degree of a node, *n* is the total number of the nodes in the network.
2. Betweenness Centrality: Betweenness centrality quantifies “the number of times a node acts as a bridge along the shortest path between two other nodes”. It was first introduced as for measuring the control of persons on the communication in an entire network by Freeman [8]. Freeman notes that “vertices that have a high probability to occur on a randomly chosen shortest path between two randomly chosen vertices also tend to have a high betweenness”. In a diffusion process, more a node is in between the network more it is likely to participate in the diffusion process. Betweenness centrality is calculated as follows:
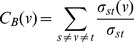
(3)where 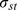 is total number of shortest paths from node s to node t and 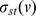 is the number of those paths that intersect node v.
3. Closeness Centrality: Connected graphs often require a metric for distance between node pairs — defined subsequently in the form of “length of shortest paths”. The farness of a node *s* is formally defined as “the sum of its distances to all other nodes”, and its closeness is defined as “the inverse of the farness” [28]. Thus, the lesser would be its total distance from other nodes, the more central a particular node will be. Closeness is considered as a temporal metric for a sequential spread of information within a network [23]. In a diffusion process, a node that has a low closeness centrality is therefore likely to receive information more quickly than others. It is calculated using the formula:
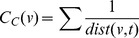
(4)where *v* and *t* are the nodes from the vertices *G*.
4. Eccentricity Centrality: The eccentricity centrality of a node is equal to “the largest geodesic distance between the node and any other node” [6]. Generally, when the Eccentricity centrality is higher for a node, the rate of diffusion for the same is lower. It is calculated as follows:
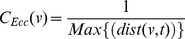
(5)where *v* and *t* are the nodes from the vertices *G*.
5. Eigenvector Centrality: It is defined as a “Measure of the influence of a node in a network”. It is calculated by assigning relative scores to all nodes in the network with the underlying idea that connections to high-scoring nodes should contribute more to the influence of the node than connections to low-scoring nodes [5]. In a diffusion process, a node with a high eigenvector centrality is connected to many such nodes connected to many other similar nodes — thereby geometrically increasing the factor governing the diffusion information in a network [5]. Eigenvector is defined as follows:
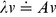
(6)where *A* is the adjacency matrix of the graph, *λ* is a constant (the eigenvalue), and *v* is the eigenvector.

## Results and Discussion

In the next sub-sections, we discuss results based on a centrality-based comparison of the network data sets under study.

### Random Networks

**Figure 6:**
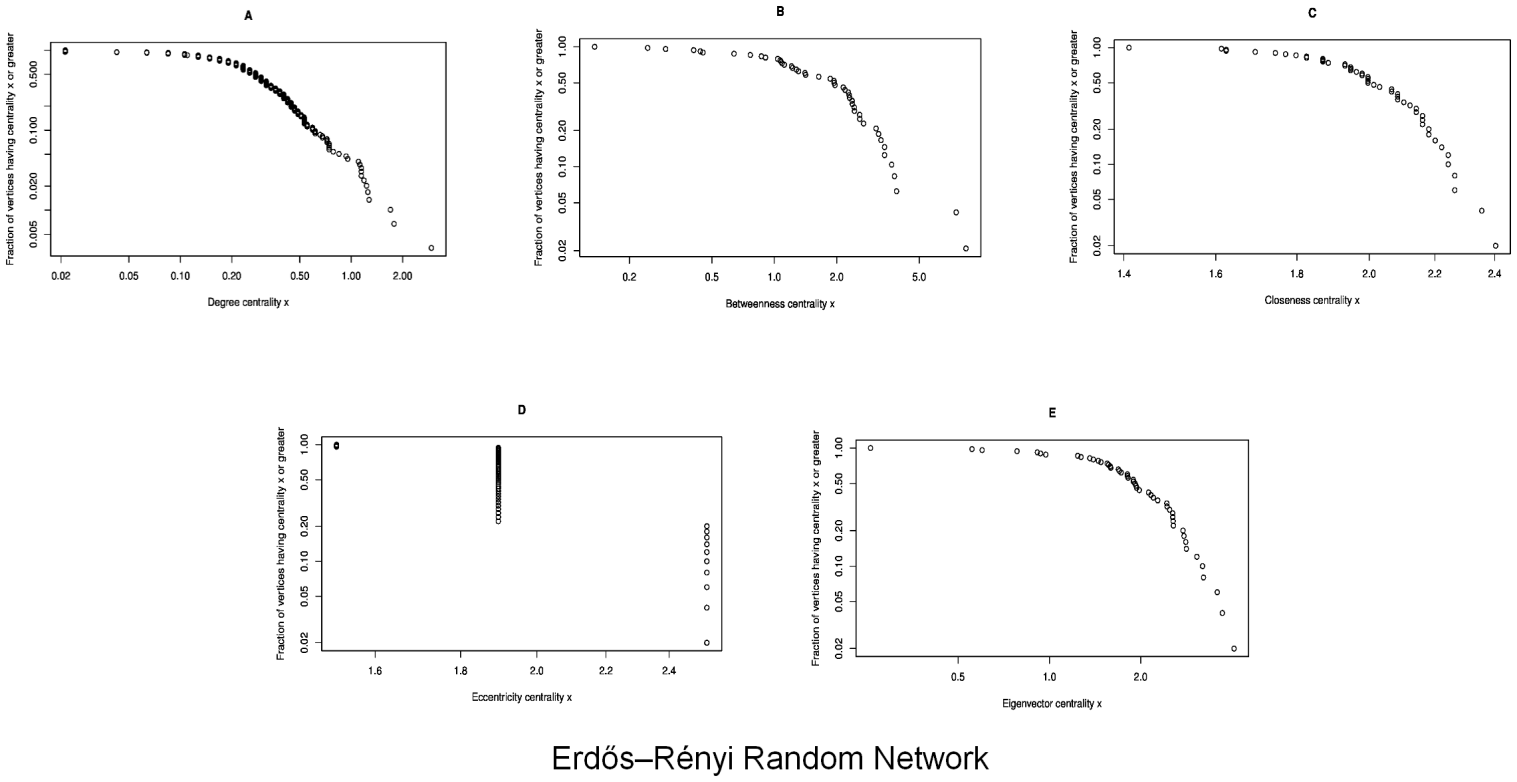
The graphs show a correlation between the frequency of the nodes and the centrality in the Erdös–Rnyi model network with *n* = 50. Figure 6a shows the Degree Centrality; there are 2 nodes having maximum value 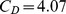 and other 3 nodes having the minimum value 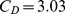. Figure 6b shows the Betweenness Centrality; there is only one node having maximum value 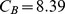 and 8 nodes having the minimum value 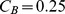. Figure 6c shows the Closeness Centrality; there is only one node having maximum value 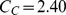 and one node having the minimum value 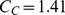. Figure 6d shows the Eccentricity Centrality; there are 9 nodes having maximum value 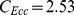 and 3 nodes having the minimum value 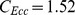. Figure 6e shows the Eigenvector Centrality; there is only one node having maximum value 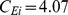 and only one node having the minimum value 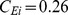. (a) Degree Centrality. (b) Betweenness Centrality. (c) Closeness Centrality. (d) Eccentricity Centrality. (e) Eigenvector Centrality.

In this Erdös-Rnyi randomly generated network, the links between the nodes are connected with a probability 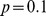. Following are observed centrality measures effects on the network:

1. Degree Centrality: We first calculate the degree centrality for the randomly generated network using equation 2 and shown in figure 6a. In figure 2a, we see the network nodes scaled and colored according to the values calculated through the degree centrality equation. Here, we see node 31 and 46 having the largest size and colored blue — indicating the highest degree centrality. Nodes with the smallest size and degree centrality are node 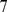 and 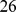, colored in red.
2. Betweenness Centrality: Likewise, the betweenness centrality has been calculated using equation 3 and is shown in figure 6b. The figure 2b shows a scaled network with node 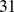 having the highest value and nodes 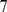 and 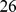 having the smallest betweenness centrality values. If we observe the results of betweenness centrality, we note that the node 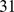 would be the most influential node in the network whereas the node 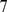 and 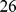 are the least influential nodes here.
3. Closeness Centrality: We have calculated the closeness centrality of the network nodes using equation 4 as can be seen in figure 6c. If we examine the scaled network in figure 2c, we can see that nodes with the lowest degree and betweenness centralities appear to have the highest closeness centrality. The closeness centrality value only has a minor variation in all nodes of this network. The node with the lowest closeness centrality is of node 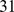 followed by node 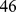. On the bases of closeness centrality, it can be seen that either node 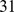 or node 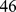 are the most influential nodes in the network.
4. Eccentricity Centrality: We calculate eccentricity centrality using equation 5 with trends which can be observed in figure 6d. In figure 2d, the scaled nodes 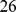, 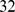 and 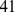 have the highest eccentricity centrality whereas the nodes 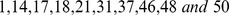 have the least centrality values clearly indicated by means of color and size for ease of visibility. The less the eccentricity centrality, is more eccentric the node is in the network. Therefore, if eccentricity is taken into account for determining the influential nodes in the network; nodes 
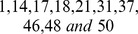
 would be considered as the most influential nodes.
5. Eigenvector Centrality: In figure 2e, the largest blue node is node 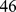 with the highest value of eigenvector centrality and the smallest red colored node is node 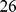 with the lowest value. The calculations are based on equation 6 with the trends displayed in figure 6e. This centrality indicates node 46 as the most important node in the network.

### Zachary's Karate Club Network

In their paper [35], the authors showed how the social friendship network of 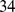 people. In the network, Mr. Hi is the club instructor and Mr. John A is the club president represented as either node 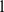 or node 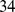 whereas the rest of the nodes are the officers. Mr. Hi and Mr. John A hold major positions in the network as they are responsible for information flow in the network therefore either of them acts as a source node. On analyzing, we see the nodes which play important roles in the network also have significant centralities values as detailed below.

**Figure 7:**
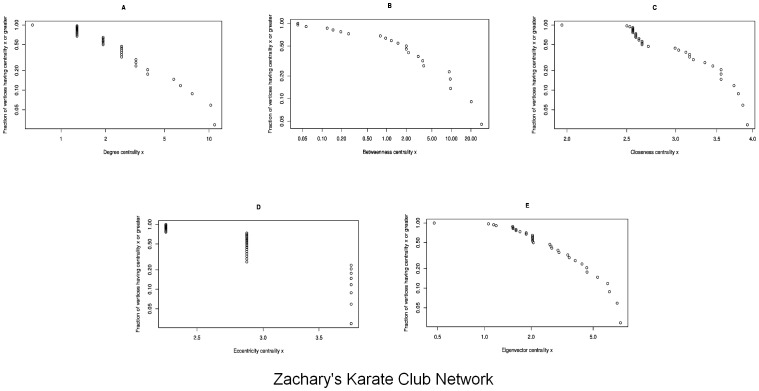
In the Zachary's Karate Club network, the graphs show a correlation between the frequency of the nodes and the centrality in the karate club network with *n* = 34. Figure 7a shows the Degree Centrality; there are 2 nodes having maximum value 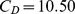 and only one node having the minimum value 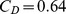. Figure 7b shows the Betweenness Centrality; there is only one node having maximum value 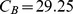 and 19 nodes having the minimum value 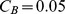. Figure 7c shows the Closeness Centrality; there are 7 nodes having maximum value 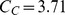 and one node having minimum value 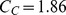. Figure 7d shows the Eccentricity Centrality; there are 8 nodes having maximum 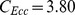 and 9 nodes having the minimum value 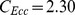. Figure 7e shows the Eigenvector Centrality; there are 2 nodes having maximum value 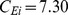 and only one node having the minimum value 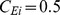. (a) Degree Centrality. (b) Betweenness Centrality. (c) Closeness Centrality. (d) Eccentricity Centrality. (e) Eigenvector Centrality.

1. Degree Centrality: In the figure 7a, the degree centrality has been calculated via equation 2. The scaled network in figure 3a shows the node 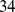 to have the highest degree centrality where as the node 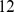 has the lowest value.
2. Betweenness Centrality: In terms of the betweenness centrality of this network, using equation 3, we can see the centrality measures shown in figure 7b. The scaled network can also be seen in figure 3b with the largest node identified as node 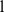, also highlighted in blue color followed by node 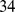. There are 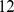 nodes whose betweenness centrality measured is the least in the network, indicated by their small sizes.
3. Closeness Centrality: Closeness centrality for each node in the network can be calculated via equation 4 and shown in figure 7c. The scaled network in figure 3c highlights the largest node in blue color identified as node 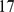 whereas node 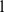 is the smallest node in red color with the lowest value of the calculated centrality. The network nodes values are distributed uniformly over the network and vary only with a slight difference among each other.
4. Eccentricity Centrality: The eccentricity centrality of the network has been calculated using equation 5 and can be observed in figure 7d. The scaled network in figure 3d, shows that nodes vary only slightly over the network. It can be observed that there are 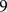 nodes in the network which have the highest values of eccentricity centrality where as node 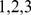 and 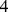, each node has the least centrality value; shown in red color.
5. Eigenvector Centrality: Based on equation 6; the centrality trend can be observed in figure 7e. We see the network layout in figure 3e with nodes scaled according to their eigenvector centrality. It can be noted here that the largest node is node 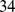 followed by node 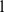 whereas the smallest node is node 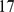, shown in red color.

### Dolphin's Social Network

**Figure 8:**
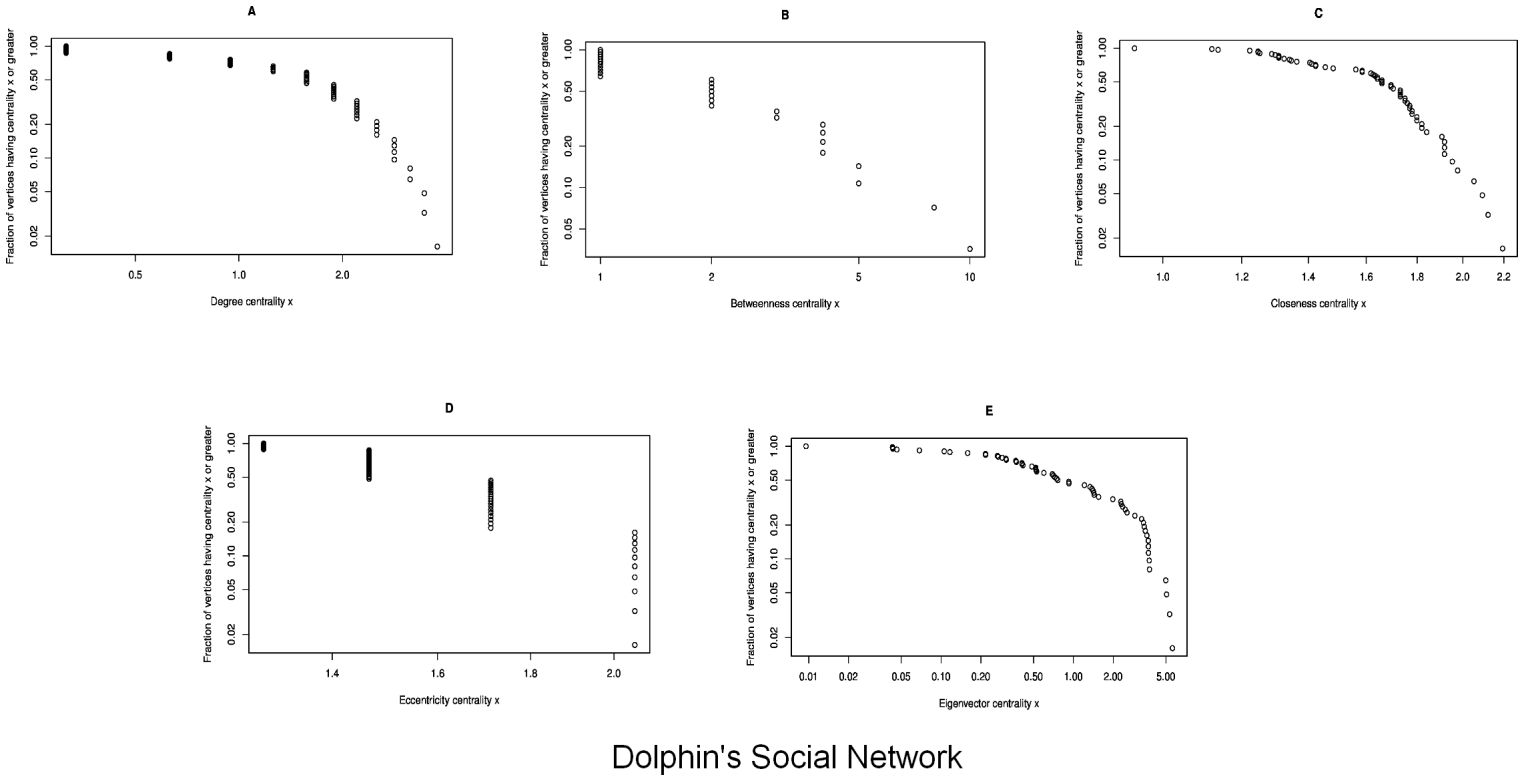
In the Dolphin Social Network, the graphs show a correlation between the frequency of the nodes and the centrality in the dolphin social network with *n* = 62. Figure 8a shows the Degree Centrality; there is only one node having maximum value 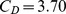 and 9 nodes having the minimum value 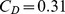. Figure 8b shows the Betweenness Centrality; there is only one node having maximum value 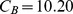 and 22 nodes having the minimum value 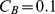. Figure 8c shows the Closeness Centrality; there are 4 nodes having maximum value 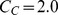 and only one node having the minimum value 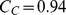. Figure 8d shows the Eccentricity Centrality; there are 10 nodes having maximum value 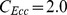 and 8 nodes having the minimum value 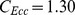. Figure 8e shows the Eigenvector Centrality; there is only one node having maximum value 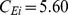 and 22 nodes having the minimum value 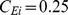. (a) Degree Centrality. (b) Betweenness Centrality. (c) Closeness Centrality. (d) Eccentricity Centrality. (e) Eigenvector Centrality.

Analysis has been performed based on the data set supplied by [20]. Details of the information regarding the nodes identity has not been provided by the authors and also the data sets provided contradicts with the network used in the paper. Following are the centralities observed on the network:

1. Degree Centrality: We have calculated the degree centrality of the network using the equation 2, its trend is shown in figure 8a. It can be observed in figure 4a, the highest degree centrality is of the node Grin whereas there are 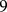 nodes (Cross, Five, Fork, MN23, Quasi, SMN5, TR82, Whitetip, Zig) which have the smallest centrality values.
2. Betweenness Centrality: Based on the equation 3, we calculate the centralities shown in 8b. Here, we note the largest sized node, SN100 having the highest centrality value as shown in the figure 4b. It is observed that all nodes having the least degree centrality values also appear to have the least betweenness centrality values.
3. Closeness Centrality: Based on the equation 4, figure 8c shows the closeness centrality values. Figure 4c shows node Zig has the largest size in the network where as the SN100 has the smallest size, clearly indicating the highest and lowest values of the centrality calculated for this network. In previously calculated centralities, SN100 has the highest values of degree and betweenness centralities.
4. Eccentricity Centrality: By using equation 5 for calculating eccentricity of the nodes in the network, we see the centrality plotted in figure 8d. Whereas in the scaled network in figure 4d, the nodes Cross, Five, Fork, TR88, TR120, TSN83, SMN5, Whitetip and Zig have the highest values indicated by their blue color whereas Beestratch, DN63, Knit, Number1, Oscar, PL, SN100, SN89, SN9 and Upbang have the least values of centralities shown as smallest in size and red in color.
5. Eigenvector Centrality: Plotted in figure 8e and shown in figure 4e, we note that the largest node in blue is Grin whereas the smallest node in red is Zig with the smallest value of eigenvector centrality calculated using equation 6.

### Neural Network

This data set represents a neural network of the worm Caenorhabditis elegans which is the only example with a complete profile of neural network. The paper concludes that “infectious diseases are predicted to spread much more easily and quickly in a small-world; the alarming and less obvious point is how few shortcuts are needed to make the world small” [33]. No details of the nodes identity are mentioned in the paper.

**Figure 9:**
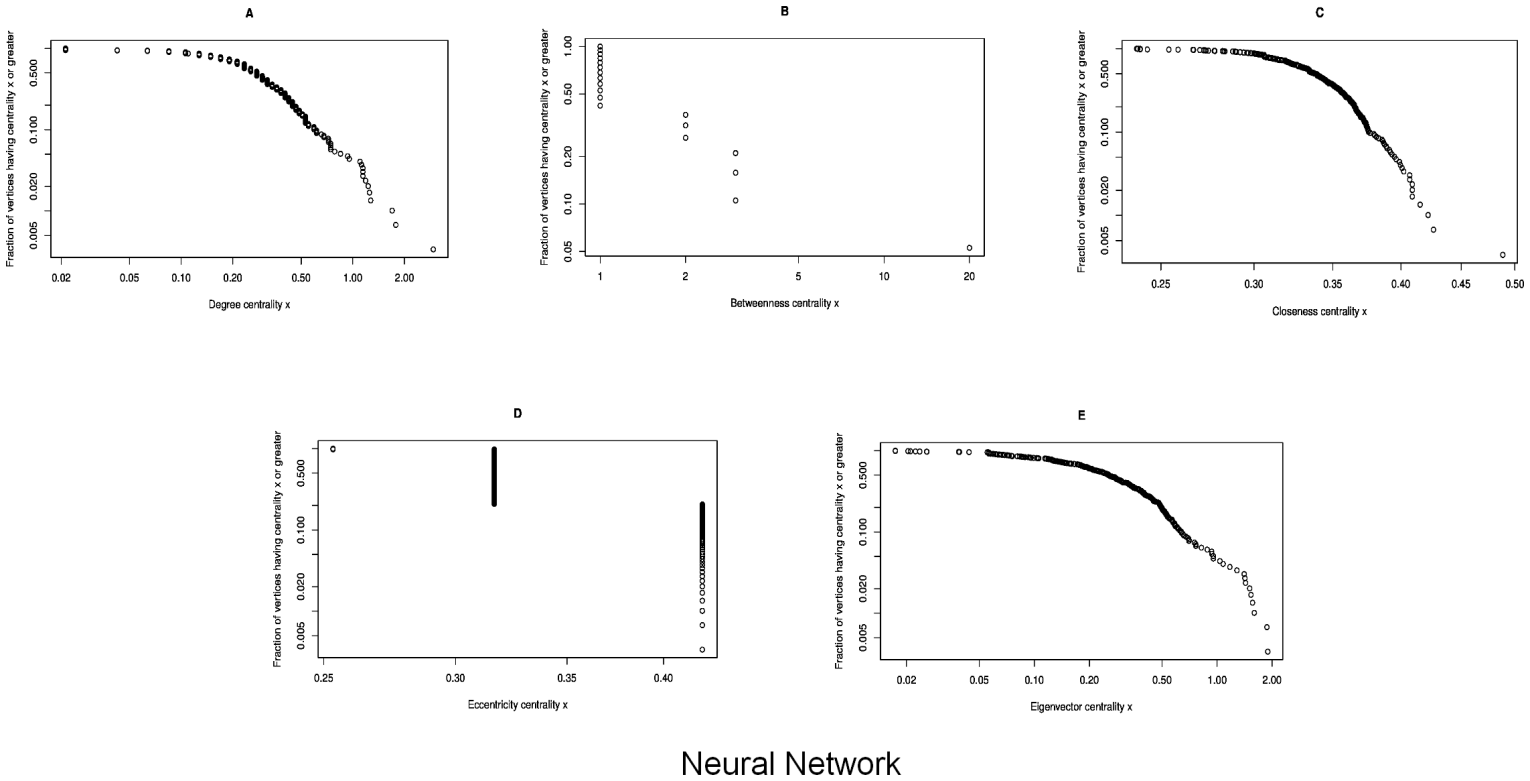
In the neural network, the graphs show a correlation between the frequency of the nodes and the centrality in the neural network with *n* = 297. Figure 9a shows the Degree Centrality; there is only one node having maximum value 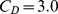 and 250 nodes having the minimum value 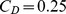. Figure 9b shows the Betweenness Centrality; there is only one node having maximum value 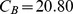 and 258 nodes having the minimum value 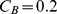. Figure 9c shows the Closeness Centrality; there are 10 nodes having maximum value 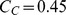 and 37 nodes having the minimum value 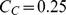. Figure 9d shows the Eccentricity Centrality; there are 60 nodes having maximum value 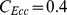 and 9 nodes having the minimum value 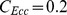. Figure 9e shows the Eigenvector Centrality; there are 13 nodes having maximum value 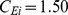 and 56 nodes having the minimum value 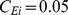.

Following are the centralities observed in the network:

1. Degree Centrality: In figure 9a, the degree centrality has been calculated through equation 2. In figure 5a, the node 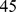 has the highest degree centrality and is thus represented as the largest node in the network. Whereas there are more than one node whose degree centrality measures are minimum therefore they are represented in red color occupying smallest size in the network. If degree centrality is observed then it is concluded that node 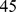 is a major node in the network.
2. Betweenness Centrality: In figure 9b, the betweenness centrality is calculated through equation 3. Here the largest sized node is node 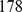 having the highest value among other nodes as shown in figure 4. We observe that the nodes whose degree centrality is smaller also have smaller value of betweenness centrality. Based on this centrality only, it can be concluded that node 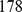 is the influential node in the network.
3. Closeness Centrality: In the figure 9c, the closeness centrality calculation is based upon equation 4. Here, the node 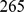 has the highest value and is thus represented as the largest node in the network as shown in figure 5c. Nodes 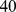, 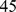 and 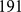 each have the least values of this centrality and are thus represented as smaller nodes.
4. Eccentricity Centrality: In the figure 9d, the eccentricity centrality has been calculated using the equation 5. Here, the highest valued node is node 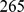. This is shown as the largest node in figure 5d, whereas the smallest nodes have least values of eccentricity — nodes 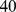, 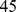 and 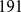.
5. Eigenvector Centrality: Next, in the figure 9e, the centrality calculations are based on the equation 6. Here, node 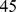 has the highest value and is thus represented as the largest node in the figure 5e. Whereas there are more than one node which have the least Eigenvector centrality values and are shown in figure 5e as the smallest nodes in the network.

### Summary of Discussion

Our analysis shows that each of the centrality measures has a unique effect on the analysis of the nodes in the network. By definition, both Closeness and Eccentricity centralities indicate the reachability of various nodes in the network. Closeness and Eccentricity centralities are similar but the Closeness centrality utilizes minimum distance from a target node to all the other nodes in the network whereas the Eccentricity centrality gives the maximum geodesic distance from the target node to all other nodes. While calculating these centralities, we have noted that the nodes which have a higher Closeness centrality also have a high Eccentricity centrality. Same is the case with Degree centrality and Eigenvector centrality, thus nodes having a high Degree centrality also have a high Eigenvector centrality. However, Betweenness centrality varies according to the topology with no noticeable or regular patterns.

In other words, we can note that there is a need to take centrality measures with a grain of salt. Not all centralities are created equal. And even if centrality measures point out important nodes in a network, this does not necessarily mean that the nodes will always be important — especially if the subjects are human users and the results can lead to serious consequences on their lives or in general, the economy of a country.

## Conclusions and Future Work

In this paper, we have carried out experiments to deduce the effects of centrality metrics for validating the roles of nodes in complex networks. We focused on the network structure whereas there are various studies which require complete influential factors or related actions of the nodes for analysis [14], [15]. Our exercise has demonstrated that such approaches however are not easy to implement in practice. For example, two of the analyzed data sets i.e. dolphins social network and neural network were published without full details of the identity of nodes in the networks.

This paper contributes by providing first steps towards a methodological validation of centrality metrics using published data sets for finding out the influence of various network nodes. The results shown by our experiments are interesting and lay the ground for further investigation. Experiments demonstrate that Eigenvector and Eccentricity centralities play a more role in determining central nodes.

Inferred concepts based on the experiments conducted in this study are summarized in table 1. In the future, the work can be further expanded and formalized to use verification and validation ideas from the domain of multiagent systems to develop a framework for performing validation of network centralities. We also foresee the use of other measures for the evaluation of important nodes in the case where nodes might be part of inter-dependent networks [32].

**Table 1:** Centralities Effect on Information Diffusion.

Centralities	Effects on information diffusion
Higher degree centrality	Diffusion rate increases and become skewed
Higher betweenness centrality	Diffusion rate increases
Higher closeness centrality	Diffusion rate decreases
Higher eccentricity centrality	Diffusion rate decreases
Higher eigenvector centrality	Diffusion rate increases
