# Correction: Towards a Methodology for Validation of Centrality Measures in Complex Networks

**DOI:** 10.1371/journal.pone.0098379

**Published:** 2014-05-16

**Authors:** The PLOS ONE Staff

The image and legend for Figure 1 are incorrect. The publisher apologizes for the error. Please see the correct Figure 1 here.

**Figure 1:**
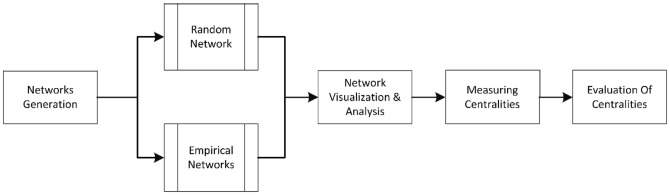
Methodology Pipeline.
